# Supplementary material for: User Perceptions of a Large Language Model–Based Chatbot for Cognitive Reappraisal of Stress: Mixed Methods Feasibility Study
Source: JMIR Hum Factors. 2026 Oct 22;13:e89736. doi: 10.2196/89736 (PMC13717329; doi:10.2196/89736)
Supplement: Supplementary file 1 [file humanfactors-v13-e89736-s001.docx]

### Multimedia Appendix 1

### Section A: System Prompts for Implementing the Chatbot

We used Azure OpenAI’s GPT-4o with a temperature setting of 0.7 and a max_tokens limit of 1024 for all API calls. Following prior work, we designed a structured prompt that was reflective and instructional [[1]](https://paperpile.com/c/7mkH9p/NL21). The prompt first assessed whether the participant’s input required a follow-up, then generated an initial response, evaluated how closely this response aligned with the expected step in the reappraisal process (as outlined in Table 1), and finally produced a revised message if needed. Since the prompt generated multiple outputs, we added formatting constraints by instructing the model to wrap each output in specific tags—for example, <Clarification Begins> and <Clarification Ends> to indicate whether a clarification was needed, and <Revised Message Begins> and <Revised Message Ends> to denote the final chatbot response in each step. The chatbot was programmed to automatically regenerate the output if the required formatting tags were missing. We also added explicit instructions to avoid common LLM idiosyncrasies observed during initial testing (e.g., ensuring that the final message is not enclosed within quotation marks).

**Prompt for Generating the First Chatbot Message**

This message will be sent by the chatbot, and it will initiate the conversation:

Welcome! I am a chatbot designed to help you reflect on your workplace stress scenarios. Together, we'll explore the situations that cause stress, identify the challenges you face, and consider different ways of thinking about these challenges.

Follow the [Instructions] below to adapt the opening message.

[Instructions]

Ensure that the opening message is appropriate. Follow these steps below.

Step 1: Analyze and Generate

Immediately craft an appropriate opening message.

Step 2: Evaluate and Refine

Reassess the initial message and look for ways to improve it.

Step 3: Finalize and Present

Finalize the refined opening message. Write only the final revised message, enclosed within specific tags to facilitate easy extraction:

<Revised Message Begins>

Final revised chatbot response. This message should not be enclosed within inverted commas.

<Revised Message Ends>

**Prompt for Responding to Each User Message (Except the Last One)**

Respond to the last user message, reflecting on the entire conversation so far and deciding on whether to move to the next theme or stay on the current theme. The response should stay within these themes and focus on promoting reflection from the user. Follow the [Instructions] below.

[Instructions]

Ensure that the response to the user message is appropriate. While generating the response, the chatbot should also decide on the following considerations based on the communication traits:

[Considerations]

Determine if the upcoming message needs to include examples or specific instructions to aid user understanding or response.

Review previous chatbot responses to decide if adjustments are needed to avoid repetition and ensure the relevance and freshness of the upcoming dialogue.

Review previous responses to see whether the user already responded to the next theme.

Check out these values before generating a response: Theme Index = [[Variable indicating which step the chatbot is in]]. Next Theme is: [[Variable indicating the next question]]. Current Theme is: [[Variable indicating the current question]].

Step 1: Deciding on clarification

Review the last user message to determine if it needs additional clarification in relation to the current theme. If the user poses a clarifying question, indicates difficulty in responding, or only partially answers the question, further clarification should be provided. First, only respond with Yes or No, enclosed within specific tags to facilitate easy extraction:

<Clarification Begins> Yes/No [It should always be No if Question index is 0] <Clarification Ends>

Then, explain the reasoning behind the decision:

<Clarification Explanation Begins> Explanation of the reason. <Clarification Explanation Ends>

Step 2: Analyze and Generate

Examine the user's last message to understand the context and requirements. If further clarification is needed (see step 1), focus on the current theme, otherwise, transition to the next theme. Immediately craft an initial response that aims to adhere to the selected theme.

Step 3: Evaluate and Refine

Reassess the initial response and justify why it does not align with the selected theme. Look for ways to improve the response.

Step 4: Finalize and Present

Finalize the refined chatbot response. Write only the final revised chatbot response, enclosed within specific tags to facilitate easy extraction:

<Revised Message Begins>

Final revised chatbot response. This message should not be enclosed within inverted commas.

<Revised Message Ends>

**Prompt for Responding to Last User Message**

Respond to the last user message reflecting on the entire conversation so far, summarize the progression of the conversation from the beginning to the end, and *clearly* state that this concludes the structured part of the conversation. Inform the user that they may choose to continue engaging in an open-ended discussion or proceed to the next section of the survey.

### Section B: Participant Demographics and PSS Scores

Table 4: Distribution of participants based on age groups

| Age Group | Count |
| --- | --- |
| 18-25 | 4 |
| 26-35 | 18 |
| 36-45 | 33 |
| 46-55 | 28 |
| 56-65 | 15 |
| 66+ | 2 |

Table 5: Distribution of participants based on job roles

| Job Role | Count |
| --- | --- |
| Software development/engineering | 33 |
| Sales/business development/strategy | 17 |
| Product management | 12 |
| Customer support | 8 |
| Finance/accounting | 5 |
| IT/infrastructure | 5 |
| Administrative/operations | 4 |
| Data science/analytics | 4 |
| Marketing/communications/public relations | 2 |
| Design/UX/UI/creative 2 | 2 |
| Research 1 | 1 |
| Other | 7 |

Table 6: Distribution of participants by PSS Scores

| PSS Score Range | Interpretation | Count |
| --- | --- | --- |
| <14 | Low Stress | 1 |
| 14-26 | Moderate Stress | 89 |
| >26 | High Stress | 10 |

### Section C: Example Conversation Between the Chatbot and a User

Table 7: Example Conversation Between the Chatbot and a User (Adapted from Real Interactions)

| Q# | Chatbot Message | User Response |
| --- | --- | --- |
| 1 | Welcome. I am a chatbot that can help you think through a work stress situation. Let us begin. What is the situation? Please describe it with as much detail as you like. | Hi, I work on a small team and the boss keeps moving the finish line. Like one day she says ok and next day she wants extra stuff. Makes me crazy. |
| 2 | Thank you for sharing that. Which part of this situation bothers you the most? | The extra stuff. She adds more every time. |
| 3 | When that happens, what are you saying to yourself in your mind? | I tell myself I gonna miss the due date and look silly. |
| 4 | I understand. Out of all the thoughts you notice, which one feels the worst? | That I look silly for sure. |
| 5 | How do you feel in your body or emotions when you think you will look dumb? | Heart beating fast, hands cold, just feel scared. |
| 6 | When you feel that way, what do you do or what do you avoid doing? | I stop talking to boss, just try fix things alone, skip lunch. |
| 7 | Let us bring these parts together. Please write a short line in this format: Trigger → Thought → Feeling → Action or Avoidance. | Trigger: boss adds more, thought: I look silly, feeling: scared, action: hide and overwork. |
| 8 | Does the trigger by itself fully explain how strong your feeling is, or might something else be adding to it? | umm… not sure what you mean |
| Follow-up | No problem. I will explain. I am asking whether the simple fact that your boss adds extra tasks is enough to create that very strong fear, or whether part of the fear comes from how you interpret her request. What do you think? | Ya maybe I take it too personally. Could be normal change, not attack on me. |
| 9 | If you looked at the extra tasks as a normal challenge instead of a personal attack, how might that change your reaction? | Maybe I ask her for plans earlier and not freak out so much. |
| 10 | What new point of view could help you see these changes as chances to grow? | Show I can adapt fast, learn better planning |
| 11 | If you keep that new point of view next time, how might your actions be different? | I will talk to boss sooner, set clear steps, and take lunch break. |
| Concluding Message | You noticed that sudden new tasks from your boss trigger the thought “I will look silly,” which brings fear, leads you to hide, and makes you work too many hours. You also saw that part of the fear comes from taking the change personally. By treating the change as a normal challenge, you could ask for clearer plans earlier, keep a calm mind, and protect your breaks.  This concludes the structured part of our conversation. You may continue with an open discussion or move to the next section of the survey. | — |

(a)

(b)

(c)

Figure 2. Screenshot examples of the chatbot conversation interface used in the study.

### Section D: Design of LLM Stress Rater

LLMs are increasingly employed in an “LLM-as-a-judge” paradigm to evaluate complex and open-ended constructs, including emotional expression, language generation quality, factual accuracy, and the dynamics of mental health conversations [[2–5]](https://paperpile.com/c/7mkH9p/X2ll+7WKA+IiWx+EiKN). Recent studies show that GPT-family models can be promising in producing ratings that correlate strongly with expert human judgments while substantially reducing annotation time and cost [[2,6]](https://paperpile.com/c/7mkH9p/X2ll+b29J). Building on prior work in psychology, HCI, and NLP, we note that stress can manifest through (1) Semantic content—explicit expressions of worry, frustration, or inability to cope, often tied to identifiable stressors [[7,8]](https://paperpile.com/c/7mkH9p/hRUq+wgJz); (2) Lexical and syntactic style—word choice, sentiment polarity, and sentence structure [[9–11]](https://paperpile.com/c/7mkH9p/54mj+t6nd+U818); and (3) Pragmatic and conversational behavior—interaction patterns such as reassurance-seeking, avoidance, or use of intensity markers [[12,13]](https://paperpile.com/c/7mkH9p/sCaX+Nim0).

The definitions of these aspects and their associated stress levels in the prompt were iteratively developed through review of prior literature and testing on example conversations. Each level was anchored with concise cues and example utterances. The prompt instructed the model to provide a short reasoning first, and then output a stress score. While our design process did not follow the formal rigor of constructing a clinical-grade rating scale, it provided a practical and interpretable complement to existing classifiers (e.g., RoBERTa-based methods [[14]](https://paperpile.com/c/7mkH9p/CCzz)). The LLM rater offered interpretable justifications tied to explicit anchors, capturing fine-grained changes in expressed stress that aligned well with our tertile-level trajectory analysis.

Below is the prompt used to elicit stress ratings using GPT-4o.

PURPOSE
Analyze the provided user text to assess the user’s psychological stress level in this segment alone. Use only the text you receive here.

DEFINITION OF PSYCHOLOGICAL STRESS
Psychological stress is a feeling of emotional strain and pressure that arises when a person perceives a situation as overwhelming or beyond their coping abilities.

Identify it through:

1. Semantic content: explicit worry, anxiety, frustration, anger, helplessness, feeling overwhelmed, tense, or inability to cope; direct mentions of stressors such as work pressure, deadlines, interpersonal conflict, financial strain.
2. Lexical and syntactic style: absolutist words like "always" and "never"; higher frequency of negative sentiment vocabulary; heavy first-person focus ("I", "my"); short, abrupt, or fragmented sentences.
3. Pragmatic and conversational behavior: repeated apologies or reassurance-seeking; mentions of overthinking; abrupt topic shifts or avoidance; intensity markers such as "!!!" or ALL CAPS or clipped phrasing like "No. Just. Go."

IMPORTANT DISTINCTION
 Do not confuse linguistic emphasis with emotional stress. ALL CAPS or punctuation can signal intensity, but interpret these within the broader emotional context of the user’s text.

STRESS RATING SCALE
 1 = No observable stress
 2 = Minimal stress
 3 = Low stress
 4 = Moderate stress
 5 = High stress

EXEMPLARS FOR STRESS LEVELS

Level 1 – No observable stress
 Cues: Neutral or positive tone, descriptive or procedural, no strain indicators.
 Example A: "Finished the report. I will send it tomorrow. Anything else you need?"
 Example B: "The meeting was fine. I noted the follow ups and put them on my calendar."

Level 2 – Minimal stress
 Cues: Mild unease or friction, limited negative words, composed phrasing.
 Example A: "A bit confused about the timeline, but I think I can sort it out."
 Example B: "I was slightly annoyed by the reschedule, though it is manageable."

Level 3 – Low stress
 Cues: Clear worry or irritation, more negative vocabulary, some first person focus.
 Example A: "I am worried the deadline is tight and I might slip if requirements change again."
 Example B: "I feel irritated because my part keeps shifting and I do not know what to prioritize."

Level 4 – Moderate stress
 Cues: Salient anxiety or overwhelm, absolutist words may appear, clipped or fragmented phrases.
 Example A: "I feel overwhelmed. Everything is urgent and I cannot keep up the pace."
 Example B: "They never give clear specs. I am tense and second guessing every decision."

Level 5 – High stress
 Cues: Strong distress, helplessness, difficulty coping, intensity markers (e.g., ALL CAPS, '!!!'), derailment.
 Example A: "I cannot do this. I am failing and it is all collapsing. I want to shut down."
 Example B: "Every day is a crisis. I feel panic and my mind goes blank during messages."

OUTPUT REQUIREMENTS
 Output ONLY in this format:

<Rating>

<Reasoning>TEXT</Reasoning>

<Stress>INT</Stress>

</Rating>

### References

1.	[Madaan A, Tandon N, Gupta P, Hallinan S, Gao L, Wiegreffe S, et al. Self-refine: Iterative refinement with Self-feedback. Oh A, Naumann T, Globerson A, Saenko K, Hardt M, Levine S, editors. arXiv [cs.CL]. 2023. pp. 46534–46594. Available:](http://paperpile.com/b/7mkH9p/NL21) <http://arxiv.org/abs/2303.17651>

2.	[Baysan MS, Uysal S, İşlek İ, Çığ Karaman Ç, Güngör T. LLM-as-a-Judge: automated evaluation of search query parsing using large language models. Front Big Data. 2025;8: 1611389.](http://paperpile.com/b/7mkH9p/X2ll)

3.	[Chandra M, Sriraman S, Khanuja HS, Jin Y, De Choudhury M. Reasoning is not all you need: Examining LLMs for multi-turn mental health conversations. arXiv [cs.CL]. 2025. Available:](http://paperpile.com/b/7mkH9p/7WKA) <http://arxiv.org/abs/2505.20201>

4.	[Chandra M, Sriraman S, Verma G, Khanuja HS, Campayo JS, Li Z, et al. Lived experience not found: LLMs struggle to align with experts on addressing adverse drug reactions from psychiatric medication use. In: Chiruzzo L, Ritter A, Wang L, editors. Proceedings of the 2025 Conference of the Nations of the Americas Chapter of the Association for Computational Linguistics: Human Language Technologies (Volume 1: Long Papers). Stroudsburg, PA, USA: Association for Computational Linguistics; 2025. pp. 11083–11113.](http://paperpile.com/b/7mkH9p/IiWx)

5.	[Gao M, Ruan J, Sun R, Yin X, Yang S, Wan X. Human-like Summarization Evaluation with ChatGPT. arXiv [cs.CL]. 2023. Available:](http://paperpile.com/b/7mkH9p/EiKN) <http://arxiv.org/abs/2304.02554>

6.	[Gilardi F, Alizadeh M, Kubli M. ChatGPT outperforms crowd workers for text-annotation tasks. Proc Natl Acad Sci U S A. 2023;120: e2305016120.](http://paperpile.com/b/7mkH9p/b29J)

7.	[Ilias L, Mouzakitis S, Askounis D. Calibration of transformer-based models for identifying stress and depression in social media. IEEE Trans Comput Soc Syst. 2024;11: 1979–1990.](http://paperpile.com/b/7mkH9p/hRUq)

8.	[Stress. [cited 11 Dec 2025]. Available:](http://paperpile.com/b/7mkH9p/wgJz) <https://psychology.org.au/for-the-public/psychology-topics/stress>

9.	[Du X, Sun Y. Linguistic features and psychological states: A machine-learning based approach. Front Psychol. 2022;13: 955850.](http://paperpile.com/b/7mkH9p/54mj)

10.	[Muñoz S, Iglesias CA. A text classification approach to detect psychological stress combining a lexicon-based feature framework with distributional representations. Inf Process Manag. 2022;59: 103011.](http://paperpile.com/b/7mkH9p/t6nd)

11.	[Turcan E, McKeown K. Dreaddit: A Reddit Dataset for Stress Analysis in Social Media. arXiv [cs.CL]. 2019. Available:](http://paperpile.com/b/7mkH9p/U818) <http://arxiv.org/abs/1911.00133>

12.	[Poirier RC, Cook AM, Klin CM. Read. This. Slowly: mimicking spoken pauses in text messages. Front Psychol. 2025;16: 1410698.](http://paperpile.com/b/7mkH9p/sCaX)

13.	[Starr LR. When support seeking backfires: Co-rumination, excessive reassurance seeking, and depressed mood in the daily lives of young adults. J Soc Clin Psychol. 2015;34: 436–457.](http://paperpile.com/b/7mkH9p/Nim0)

14.	[Hartmann J, Heitmann M, Siebert C, Schamp C. More than a feeling: Accuracy and application of sentiment analysis. Int J Res Mark. 2023;40: 75–87.](http://paperpile.com/b/7mkH9p/CCzz)
